# Particle morphomics by high-throughput dynamic image analysis

**DOI:** 10.1038/s41598-019-46062-6

**Published:** 2019-07-03

**Authors:** Youmin Sun, Zhengqing Cai, Jie Fu

**Affiliations:** Department of Environmental Science & Engineering, Fudan University, Shanghai, 200438 China

**Keywords:** Geochemistry, Geophysics

## Abstract

A novel omics-like method referred to as “particle morphomics” has been proposed in the present study. The dynamic images of >2,000,000 particles per sample in sediments, soils and dusts were collected by a Sympatec GmbH QICPIC particle size and shape analyzer, and the morphological descriptors of each particle including equivalent diameter, sphericity, aspect ratio and convexity were extracted as the “particle morphome”. Various multivariate analyses were adopted to process the high-throughput data of particle morphome including analyses of alpha and beta diversities, similarity, correlation, network, redundancy, discretion and principal coordinate. The outcome of particle morphomics could estimate the morphological diversity and sketch the profile of morphological structure, which aided to develop a morphological fingerprint for specific particle samples. The distribution and properties of particle assemblages of specific morphology could also be evaluated by selecting particles with respect to filter criteria. More importantly, the particle morphomics may be extended to investigate and explain the biogeochemical and environmental processes involved with particle morphology if linked with external variables.

## Introduction

The morphology, that is size and shape, is an important characteristic of solid particles which determines their many properties^1^. For instance, the size and density of fine sediment particles govern the settling velocity according to the classic Stokes’ law^2^. In terms of aerodynamics, shape has a direct impact on the drag forces acting on ash particles of floating in the air^3^. Thus, quantifying particle morphology is a fundamental issue in particle research.

Sieving is probably the most used method to determine the particle size distribution. This traditional method is time consuming and expensive, and has deviations when particle shape is involved^4^. Due to outstanding features such as fast measurement and simple operation, laser diffraction over the past 40 years has gradually become the most frequently applied method for particle size analysis^5^. However, laser diffraction technique lacks of reliable and quantitative particle shape characterization. In the last 10–20 years, image analysis is probably the mainstream approach to simultaneously characterize the size and shape of particles, which uses a two-dimensional image of a three-dimensional particle and calculates various size and shape parameters from this image^1^. Especially, the dynamic image analysis technique can capture the three-dimensional morphology of particles by rotating particles with random orientation which allows the measurement of the true characteristics of particles^6^.

Currently, particle morphological analyses focus on size/shape distribution or average information such as volume mean diameter (VMD), while the morphological diversity and structure extracted from information of millions particles have not been addressed. The heterogeneous particles or specific particle assemblages in a particle sample are also difficult to reveal using conventional morphological analyses. Moreover, the morphological information of particles is often not associated with external factors (e.g., hydraulic conditions), which veils the scientific value of particle morphology.

The development of high-throughput “omics” approaches over the last few years has considerably accelerated the pace of discovery in multidisciplinary sciences^7^, which aims at the collective characterization and quantification of pools of various information. In this paper, attempts have been made to establish an omics-like method referred to as “particle morphomics” to comprehensively analyze the large dataset of particle morphological descriptors (“particle morphome”). The particle morphomics would open up a research approach for particle morphological analyses and reacquaint the importance of particle morphology.

To this end, a set of particle samples including 7 sediments, 4 soils and 3 dusts were collected (Supporting Information (SI) Table S1). After pretreatment of natural drying, removing biological materials and rocks, and passing through a 5 mesh (5 mm) sieve, the dynamic image information of >2,000,000 particles per sample were collected by a QICPIC particle size and shape analyzer (Sympatec GmbH, Clausthal-Zellerfeld, Germany)^8^, and the morphological descriptors of each particle including equivalent diameter, sphericity, aspect ratio and convexity were extracted by the WINDOX 5 software^9^. The measurement principle of QICPIC system is described in SI Text S1. The details on the workflow of extracting data by WINDOX 5 are shown in SI Text S2. Various multivariate analyses were employed to process the high-throughput data of particle morphome including analyses of alpha and beta diversities, similarity, correlation, network, redundancy, discretion and principal coordinate. For comparison, the traditional sieving was also conducted to analyze the particle size distribution.

## Results

### Repeatability and reliability of particle size and shape analysis

According to ISO 14488 standard^10^ and instrumental property, the measure parameters of dynamic image analysis were optimized to achieve a minimum number of calculated particles per measurement of above 2,000,000 and an optical concentration of within 0.5–1% during each measurement, assuring the accuracy and reliability of measured data. Table 1 presents the mean morphological descriptors of five repeated measurements as a basis for calculating the volume of the particle. The relative standard deviation (RSD) for VMD, volume mean sphericity (VMS), volume mean aspect ratio (VMA) and volume mean convexity (VMC) was within 1.05–3.82%, indicating a good repeatability and reliability of dynamic image analysis.

**Table 1:** Volume mean morphological descriptors of five repeated measurements.

	VMD/μm	VMS	VMA	VMC	Number of calculated particles	Optical concentration/%
Measure 1	211.40	0.76	0.66	0.84	3548498	0.53
Measure 2	205.41	0.78	0.68	0.86	3323588	0.56
Measure 3	208.13	0.75	0.67	0.84	3319361	0.57
Measure 4	197.25	0.78	0.67	0.85	3699180	0.50
Measure 5	192.74	0.78	0.66	0.84	3730626	0.50
Mean value	202.98	0.77	0.66	0.84	3524251	0.53
SD	7.76	0.01	0.00	0.00	197499	0.03
RSD/%	3.82	1.83	1.25	1.05	5.60	6.14

Abbreviation: VMD, volume mean diameter; VMS, volume mean sphericity; VMA volume mean aspect ratio; VMC, volume mean convexity; SD, standard deviation; RSD, relative standard deviation. The measured sample was the fraction of No. 8 sample (SI Table S1), passing through a 65 mesh (250 μm) sieve and retaining on a 150 mesh (100 μm) sieve.

### Comparison with traditional sieving method

Typically, the VMDs calculated by dynamic image analysis and sieving method showed a good consistency (SI Fig. S1a), which was evaluated by the cross-validation correlation coefficient (*q*^2^)^11^:

1 $${q}^{2}=1-\frac{{\sum }_{{\rm{i}}=1}^{{\rm{n}}}\,{({{\rm{VMD}}}_{{\rm{d}}}-{{\rm{VMD}}}_{{\rm{s}}})}^{2}}{{\sum }_{{\rm{i}}=1}^{{\rm{n}}}{({{\rm{VMD}}}_{{\rm{s}}}-{{\rm{VMD}}}_{{\rm{mean}}})}^{2}}$$

where n is the number of samples, VMD_d_ and VMD_s_ are the VMD calculated by dynamic image analysis and sieving method, respectively, and VMD_mean_ is the mean value of VMD_s_. The obtained *q*^2^ is 0.565, indicating the sieving method is relatively reliable for acquiring the mean size information of particle samples. However, besides time consuming, the sieving method cannot provide an accurate profile of particle size distribution due to the limited size class. For instance, some small peaks in the distribution curve cannot be identified by sieving method (SI Fig. S1b), and the calculated kurtosis always showed an abnormally high value (SI Fig. S1c). Therefore, the dynamic image analysis is a more effective and accurate method to gain the morphological profiles of particle samples.

### Morphology distribution parameter and alpha diversity

Table 2 summaries the morphology distribution parameters and alpha diversity indices of the measured 14 particle samples. The expectation (mean value) of the size (equivalent diameter) ranged from 94.62 μm to 678.40 μm with a mean value of 250.15 μm. The shape of the average size distribution curve is leptokurtic and right-skewed (SI Fig. S2a), because of the positive values of kurtosis and skewness^12^. Alpha diversity is an ecological term and its original meaning is the species diversity in sites or habitats at a local scale^13^. Here we use it to indicate the particle morphological diversity in a sample. Two diversity indices, Simpson and Shannon, were employed to evaluate the variety and heterogeneity of particle morphology. Shannon index has its foundation in information theory and represents the uncertainty about the identity of an unknown individual^14^. Simpson index represents the probability that two randomly chose individuals belong to different classes^15^, and it is a dominance index because it gives more weight to dominant classes. A higher value of Simpson/Shannon index represents a more diversity of particle morphology. For particle size, the obtained Simpson and Shannon indices were 0.859–0.948 (mean value = 0.919) and 2.23–3.04 (mean value = 2.71), respectively.

**Table 2:** Morphology distribution parameters and alpha diversity indices of the measured 14 particle samples.

		X_16_	X_50_	X_84_	Expectation	SD	Kurtosis	Skewness	Simpson	Shannon
Size	Min-value	37.36 μm	87.79 μm	146.83 μm	94.62 μm	53.68 μm	0.86	0.84	0.859	2.23
	Max-value	405.88 μm	584.62 μm	883.18 μm	678.40 μm	431.50 μm	17.42	3.48	0.948	3.04
	Mean-value	93.74 μm	195.63 μm	407.71 μm	250.15 μm	191.33 μm	4.89	1.72	0.919	2.71
Sphericity	Min-value	0.57	0.73	0.85	0.72	0.08	−0.38	−1.49	0.949	3.25
	Max-value	0.76	0.86	0.91	0.84	0.13	2.50	−0.44	0.978	3.95
	Mean-value	0.64	0.79	0.88	0.77	0.11	0.35	−0.79	0.971	3.73
Aspect ratio	Min-value	0.45	0.63	0.77	0.62	0.12	−0.25	−0.69	0.972	3.80
	Max-value	0.54	0.69	0.80	0.68	0.16	0.55	−0.39	0.980	4.09
	Mean-value	0.50	0.66	0.78	0.65	0.13	−0.08	−0.48	0.970	3.96
Convexity	Min-value	0.71	0.82	0.89	0.81	0.05	0.71	−2.96	0.887	2.56
	Max-value	0.89	0.95	0.97	0.93	0.10	16.92	−0.59	0.965	3.57
	Mean-value	0.75	0.85	0.90	0.84	0.08	3.34	−1.28	0.952	3.33

Abbreviation: X_i_, the value of morphological descriptor at the cumulative distribution of percentage i (%); SD, standard deviation. The number of calculated particles ranged from 2,506,202 to 19,958,896 per sample, and the optical concentration ranged from 0.50% to 0.98%.

Sphericity is defined as the degree to which the particle similar to a smooth sphere^16^, and represents both the irregularity of particle form and roughness of particle surface. The calculated mean particle sphericity of the measured samples was between 0.72 to 0.84 (Table 2), indicating the majority of particles had a relatively regular form. Compared with size distribution, the average sphericity distribution was more close to a normal distribution (SI Fig. S2b), since the kurtosis value was close to zero^12^; the distribution curve of sphericity was left-skewed because of negative value of skewness, indicating a trend toward regular particle form. The Simpson and Shannon indices for sphericity distribution were 0.949–0.978 (mean value = 0.971) and 3.25–3.95 (mean value = 3.73), respectively. The aspect ratio is defined as the ratio of the Feret’s minimum length to the Feret’s maximum length^16^, and has been used historically to classify the general form of particles (e.g., equant, acicular, or fibrous). The shape of average aspect ratio distribution was similar with that of sphericity distribution that is close to the normal distribution and left-skewed (SI Fig. S2b), but with a larger variation because of relatively larger Simpson and Shannon indices (Table 2). The convexity is a measurement of the particle edge roughness and the particle compactness, and is defined as the ratio of the actual projection area and the convex hull area^16^. Relative higher values of convexity (expectation = 0.81–0.93) were observed in the measured samples (Table 2), indicating the measured particles generally had a high compactness. The shape of average convexity distribution was leptokurtic and left-skewed (SI Fig. S2b), and the calculated Simpson and Shannon indices were 0.887–0.965 (mean value = 0.952) and 2.56–3.57 (mean value = 3.33), respectively.

After comparing the diversity indices of the morphological descriptors, we found the shape factors, especially aspect ratio, had much higher values than equivalent diameter (Table 2), indicating a larger heterogenicity of particle shape in the samples. The distribution curves of shape factors comprised of substantial numbers of small peaks, while the distribution curve of equivalent diameter was relatively smooth with a dominant peak (Fig. S2), which is consistent with alpha diversity evaluation.

**Figure 1:**
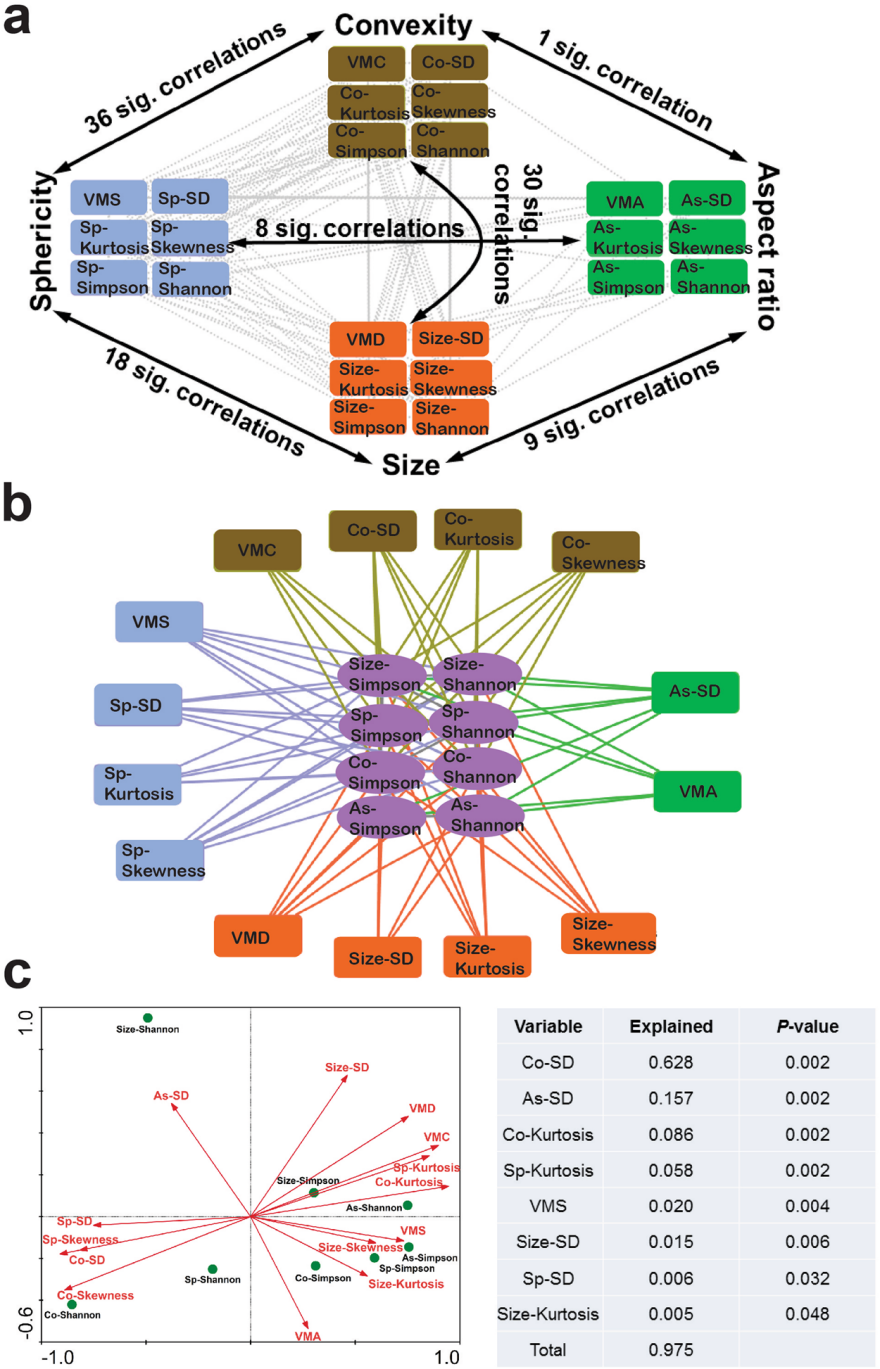
Relations between particle morphology distribution parameters and alpha diversity indices. (**a**) Network plot for significant relations among distribution parameters and diversity indices of particle size, sphericity, aspect ratio and convexity. **(b)** Network plot of the associated distribution parameters with particle morphological diversity. **(c)** Redundancy analysis (RDA) for ranking the most important distribution parameters that explained the particle morphological diversity. Each edge connecting two nodes in **(a)** and **(b)** represented a significant correlation (*p* < 0.01). Forward selection of variables was based on the Monte Carlo permutation test (n = 499). Abbreviation: VMD, volume mean diameter; VMS, volume mean sphericity; VMA volume mean aspect ratio; VMC, volume mean convexity; SD, standard deviation; sp, sphericity; as, aspect ratio; co, convexity.

Correlation analysis indicated these morphology distribution parameters and diversity indices were interconnected (SI Sheet 1). Size, sphericity and convexity factors showed strong associations with each other, while aspect ratio exhibited a relatively independent character (Fig. 1a). Most morphology distribution parameters posed significant effects on the diversity indices (Fig. 1b). Redundancy analysis (RDA) indicated convexity SD was the most important variable that explained 62.8% of the morphological diversity (Fig. 1c). Due to the strong positive correlations between convexity SD and diversity indices (SI Sheet 1), a large variety of particle convexity will be expected to result in a high morphological diversity.

### Morphology distribution comparison and beta diversity

Discrete analysis of morphology distribution at each size or shape class may provide the information on which fraction of particles showed the largest variability among the measured samples. SI Fig. S3 illustrates the discretion of particle morphology for the measured samples. Typically, the larger size (>500 μm) or lower sphericity (<0.4) of particles exhibited a larger discretion with the RSD being above 100%. Yet for aspect ratio and convexity, the particles at the two tails of the morphology distribution showed higher degree of dispersivities.

**Figure 2:**
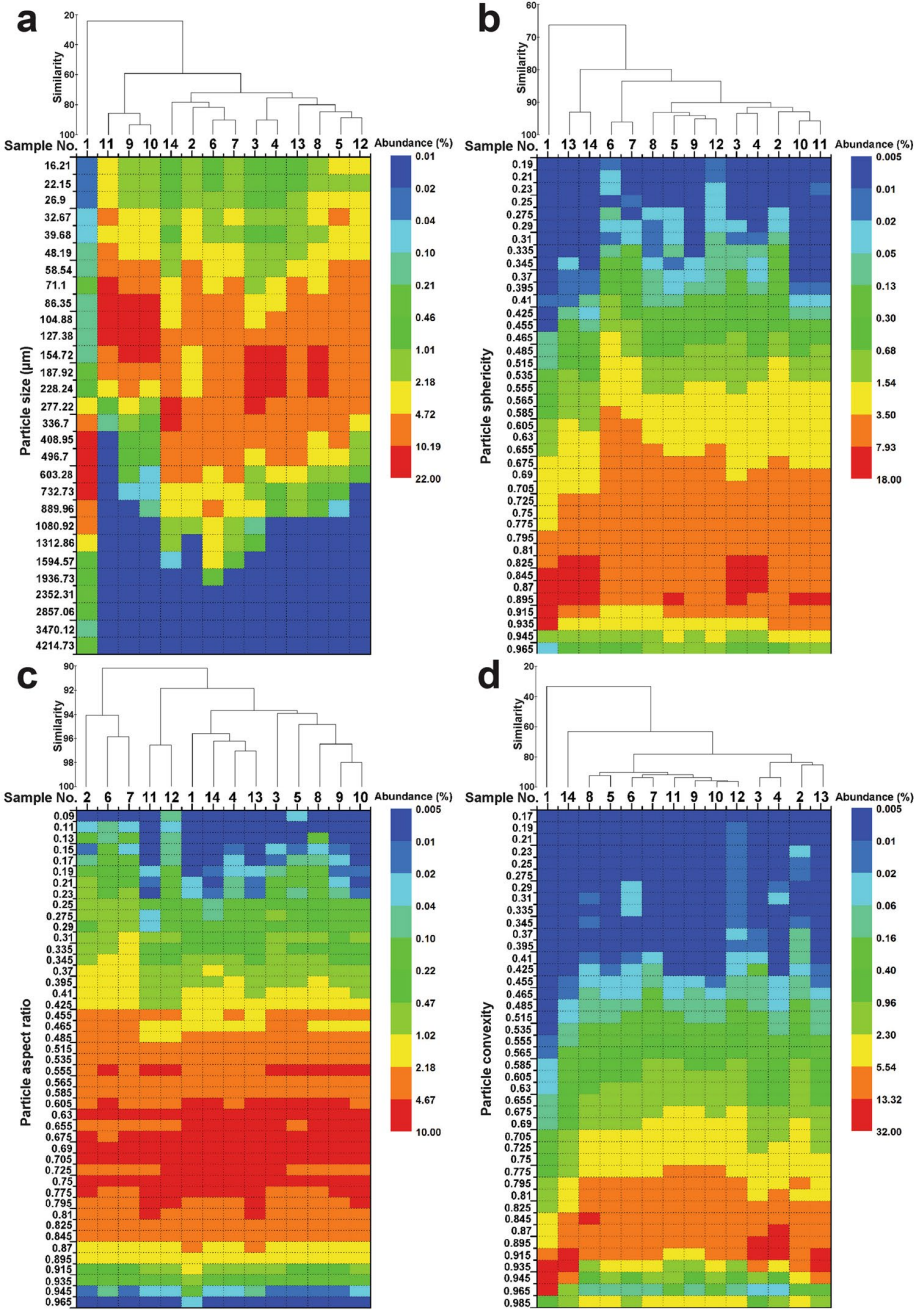
Heatmap with clustering of particle morphology distribution for the measured samples: (**a**) size, (**b**) sphericity, (**c**) aspect ratio and (**d**) convexity. Clustering of samples was conducted using the mode of group average based on Bray-Curtis similarity.

Beta diversity originally referred to the ratio between regional and local species diversity in ecology^13^, and here we used it to describe the particle diversity between a set of particle samples. Bray-Curtis similarity analysis was employed to estimate the similarity or disparity between particle samples in terms of morphology distribution. Figure 2 summaries the results of similarity analysis with the heatmap illustrating the particle morphology distribution. Of all the measured 14 samples, the similarity based on different morphological descriptors was in the order of aspect ratio (90.2%) > sphericity (66.42%) > convexity (33.27%) > size (24.23%). Therefore, the measured samples showed a large diversity in particle size and convexity, a moderate diversity in sphericity, but quite a low diversity in aspect ratio. Inspecting the individual samples, sample 1 exhibited a distinct disparity with the other samples in distributions of particle size, sphericity and convexity, and high abundant of particles of relatively larger size, sphericity and convexity were observed in sample 1.

**Figure 3:**
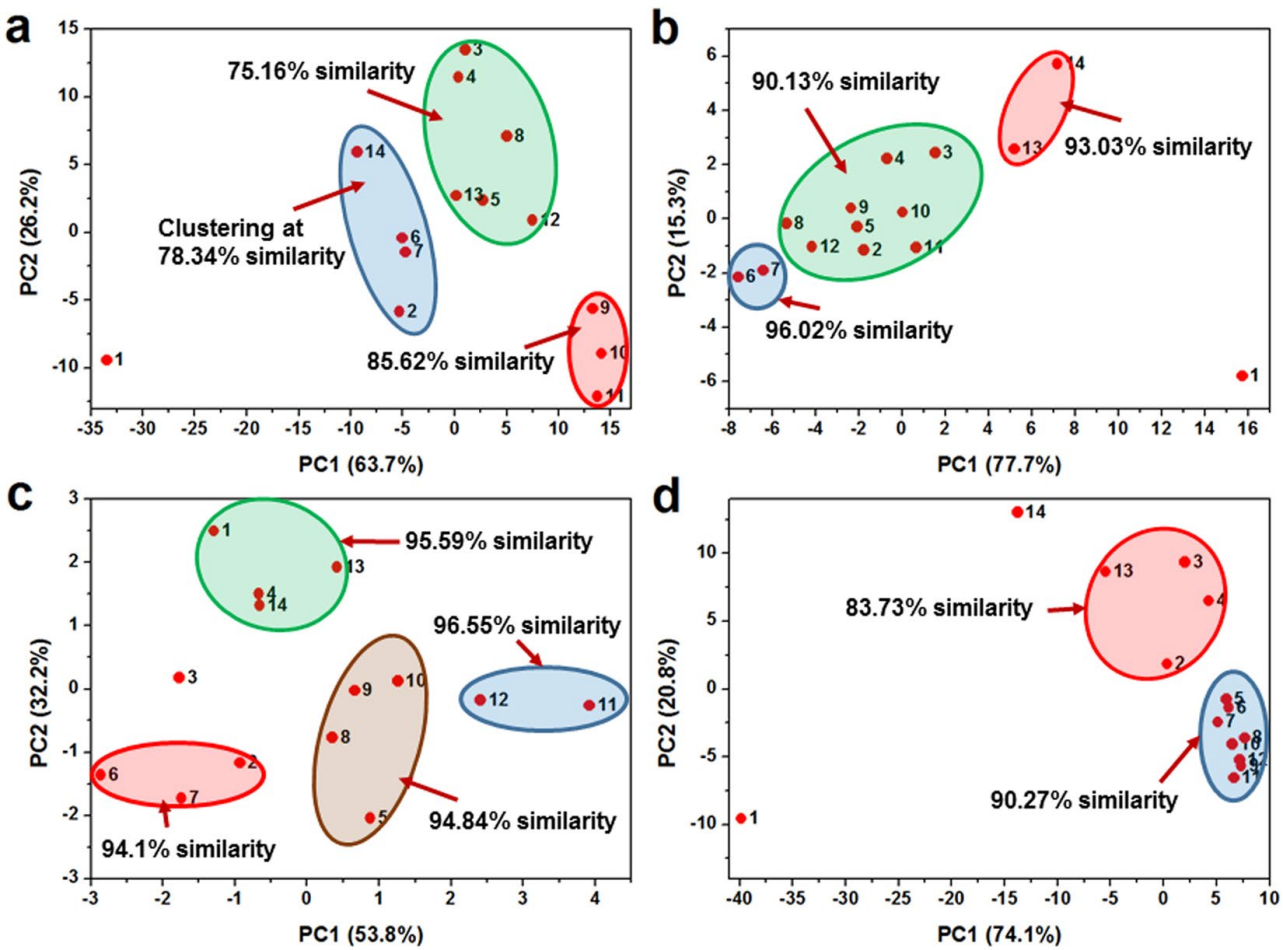
Principal coordinate analysis (PCoA) of the measured samples based on the particle morphology distribution: (**a**) size, (**b**) sphericity, (**c**) aspect ratio and (**d**) convexity. The sample No. was labelled to each sample dot. The sample cluster was circled and the similarity was calculated by Bray-Curtis similarity analysis.

Principal coordinate analysis (PCoA) diagram was further used to visually illustrate the beta diversity of particle morphology among samples (Fig. 3). The scale of coordinate axes for particle size (Fig. 3a) and convexity (Fig. 3d) was considerably greater than that for particle sphericity (Fig. 3b) and aspect ratio (Fig. 3c), indicating a much higher diversity of particle size and convexity in the measured samples. This is because that the variable matrix of large variety will be apt to produce larger eigenvalues, stretching the axis scale. The samples having similar morphology distribution will automatically aggregate to form several clusters, and the distance between samples indicates the disparity of morphology distribution. For instance, sample 6 and 7 approached each other and located at the leftmost end of PC1 axis in the PCoA diagram of particle sphericity (Fig. 3b). The two sediment samples had the same source from a small river (SI Table S1), and their particle sphericity distribution showed a very high similarity of 96.02% (Fig. 2b).

### Characterization of specific particle assemblages

It is of importance to classify and characterize the specific particle assemblages in a heterogeneous sample. For instance, the suspended particles in marine environments comprise of various assemblages such as phytoplankton-dominated particles, organic non-algal particles, and mineral-dominated particles^17^. Discriminating these specific particle assemblages will be of great help for investigations on biogeochemical processes (e.g., phytoplankton blooms)^18^, ecosystem structure (e.g., food web)^19^ and dispersion of pollutants (e.g., spilled oil)^20^ in marine environments. Since different particle assemblages have specific morphological characteristics, morphome may provide useful information for characterization of particle assemblages.

**Figure 4:**
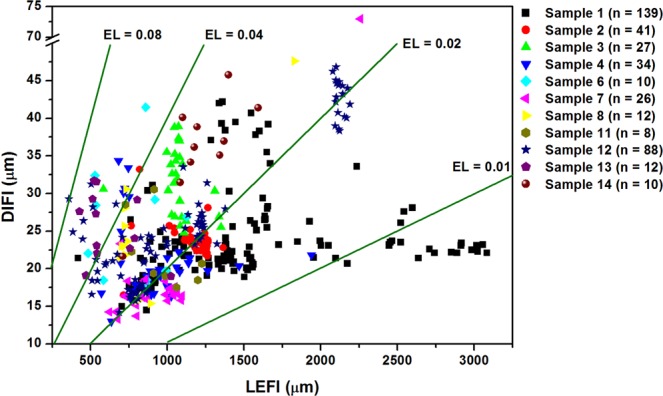
Distribution of fiber-like particles in the measured samples. No fibers were detected in samples 5, 9, and 10. LEFI = Length of fiber, DIFI = Diameter of fiber, and Elongation (EL) = DIFI/LEFI.

The WINDOX 5 software provides a function of particle selection with respect to filter criteria of particle size and shape. For exhibition, the impurity fiber particles in the measured samples were picked up under the filter condition of “aspect ratio ≤0.25 and sphericity ≤0.25 and convexity ≤0.9”. The results showed the fiber particles were very few in the samples, and only 407 fibers were identified with a mean length of fiber (LEFI) of 1.02 mm (0.36–3.08 mm), mean diameter of fiber (DIFI) of 25.47 μm (12.99–73.01 μm), and mean elongation (EL = DIFI/LEFI) of 0.028 (0.007–0.082) (Fig. 4). Typically, the soil samples (8–11) contained the least fiber particles, while sediment samples (1–7) and dust samples (12–14) had relatively higher number of fibers. The particle images (SI Fig. S4) indicated a significant difference of fiber particles in morphology and orientation in sediment samples and soil/dust samples, suggesting a distinctly different nature and source of these fiber particles. For instance, the fiber particles in sediment sample 1 were generally elongated, and lying to pass through the flow cuvette during the QICPIC measurement process. These fiber particles might come from the suspended matters in the Yangtze River, which had a relatively low density and flat surface due to the dissolution and hydraulic washing by water. However, the fiber particles in soil sample 8 and dust sample 12 were relatively thicker with irregular surface, and vertically oriented in the measured particle flow. These fiber impurities might come from anthropogenic products (e.g., man-made fibers, and plastic residues) and/or plant residual fractions (e.g., root tips, and filamentous pollen).

### Morphological fingerprints of particle samples

The fingerprint technique is to collect and analyze the profiles of chemical compositions or physiochemical properties of investigated samples by various analytical techniques to identify and compare the disparity between samples. The fingerprint technique has wide applications such as controlling the quality of herbal medicine by using high-performance liquid chromatography (HPLC)^21^, discriminating the geographical origin of food produce by using nuclear magnetic resonance (NMR)^22^, and identifying the source contribution of sediment by using compound specific stable isotopes (CSSI)^23^.

**Figure 5:**
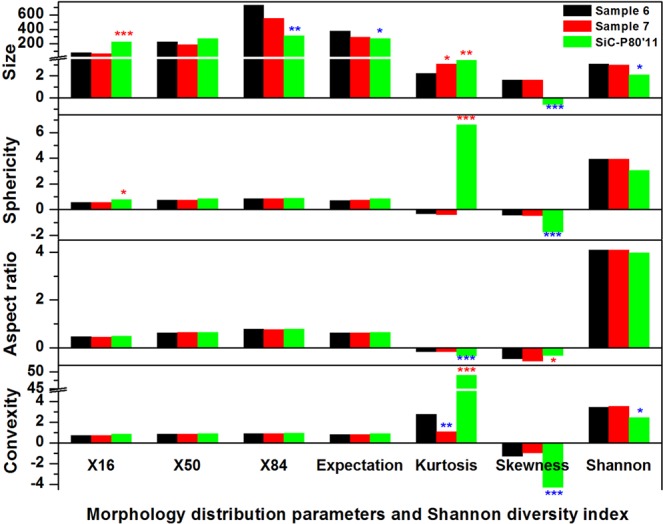
Morphological fingerprints of sediment samples 6 and 7, and silicon carbide reference material SiC-P80′11. X_i_ represented the value of morphological descriptor at the cumulative distribution of percentage i (%). Significant increases (red color) or decreases (blue color) compared with sample 6 were marked using * (by >25%), ** (by >50%), or *** (by >100%).

To visually and quantitatively compare the difference of particle samples in particle size and shape, a morphological fingerprint was constructed by adopting the morphology distribution parameters (i.e., X_16_, X_50_, X_84_, expectation, kurtosis and skewness) and Shannon diversity index. Figure 5 shows the morphological fingerprints for samples 6 and 7, and a silicon carbide reference material SiC-P80′11, which is used for re-certification of the Sympatec instrument. It is obvious that the two sediment samples 6 and 7 had a highly similarity in particle size and shape, which is due to their same source (SI Table S1). However, the morphological fingerprint of SiC-P80′11 was vastly different with that of sample 6. Generally, the SiC-P80′11 had a lower Shannon diversity index and a higher kurtosis for particle morphology, indicating a higher homogeneity in man-made SiC-P80′11 compared with the natural sediment samples. In addition, the relatively high values of shape descriptors (i.e., sphericity, aspect ratio, and convexity) for SiC-P80′11 indicate these particles had regular and equant shapes with relatively smooth edge and high compactness.

## Discussion

**Figure 6:**
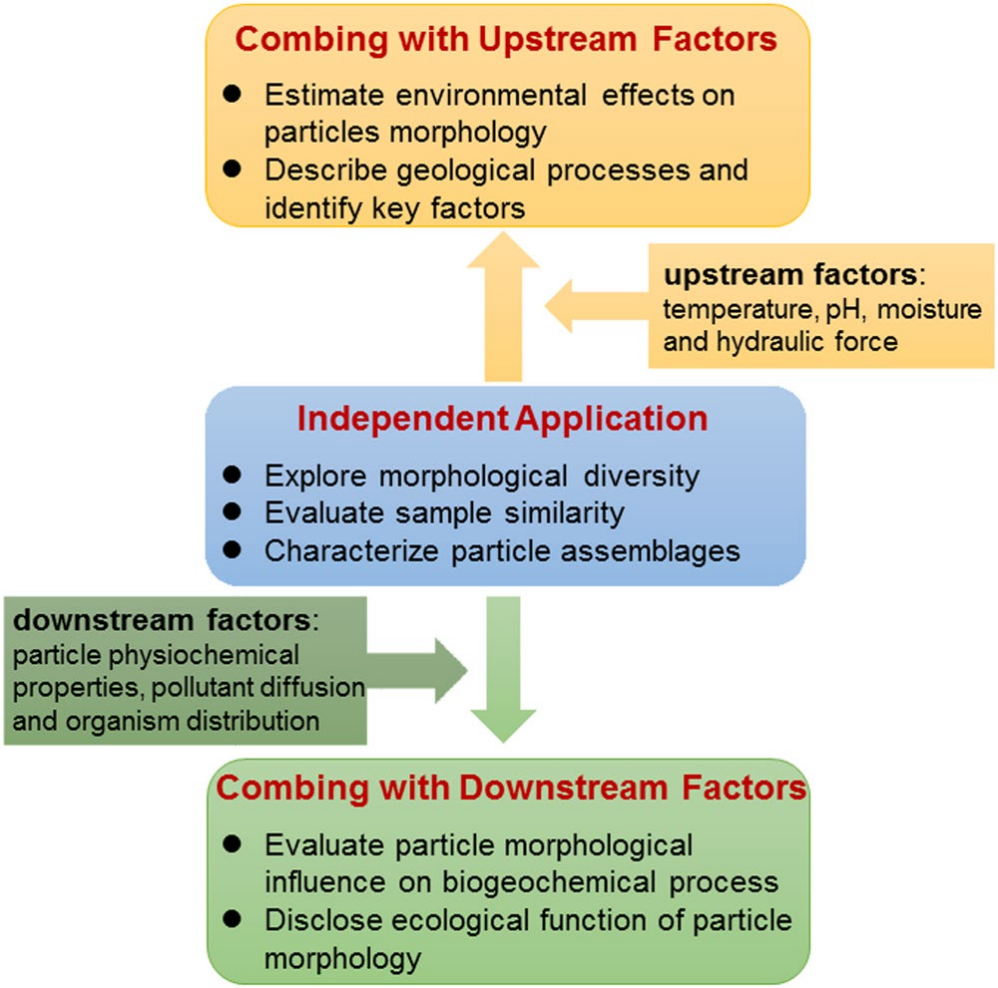
Schematic diagram for application of particle morphomics.

This paper has presented the independent application of particle morphomics. Besides, particle morphomics may play a greater role in geoscience and environmental studies when combing with the external factors (Fig. 6). When combing with “upstream factors” such as temperature, pH, moisture, and hydraulic force, particle morphomics can be used to estimate the effect of environmental factors on the shaping of particles, describe the geological processes and identify the key factors. When combing with “downstream factors” such as physical properties and chemical compositions of particles, pollutant diffusion, and organism distribution, particle morphomics can be used to evaluate the influence of particle morphological factors on the biogeochemical process, and reposition the role of particle morphology in the ecosystem function. Some potential fields to apply particle morphomics are given below:

1. **Sediment source tracing**. The excess supply of fine sediment particles from accelerated soil erosion may cause serious consequences, such as degrading riverine and coastal environments, facilitating the downstream transfer of particle-bound contaminants, and increasing the cost of operating and maintaining water treatment and transportation infrastructure^23^. Gaining the knowledge of the relative contribution of different sources supplying sediment to riverine, lacustrine and coastal systems is a crucial prerequisite to implementing efficient best practices necessary to limit the off-site impacts of excessive sediment delivery^24^. While there are many literatures published on sediment source tracing by using the various particle biogeochemical properties (e.g., fallout radionuclides, organic matter and magnetic minerals) as tracers^25,26^, few have investigated the effects of particle morphology on sediment source signatures or employed particle morphology as a tracer. In fact, all the processes of soil erosion and detachment, sediment transportation, deposition and abrasion are particle morphology dependent, and the selectivity of particle morphology acts over the entire landscape^27^. Not only are sediment transport processes particle morphology selective, the properties used to trace sediments may have different affinities (e.g. preferential adsorption/absorption) to particle morphology^28^. Particle morphomics can be a powerful tool to analyze particle morphology, and provided information can aid in understanding of particle morphology-biogeochemical tracer interactions and improving the predictability and accuracy of sediment source tracing.
2. **Volcanic ash particles research**. Volcanic ash particles carry a wealth of information on various processes occurring during volcanic eruptions, and their morphology has been recognized to reflect numerous eruptive parameters such as magma viscosity, volatile content, degree of interaction with external water, particle transport and sedimentation^3,29^. Many investigations of ash morphology have been carried out with multi-targets at unravelling the origin of ash and the related magma fragmentation dynamics^30^, determining the physical properties of magma and its degree of interaction with external water^31^, modeling the processes of ash dispersal and settling^32^, forecasting the continuation or ending of an eruptive episode^29^. The particle morphomics methodology outlined in this study can promote volcanic ash particles research to dredge up the intrinsic nature of morphology, e.g., diversity, backtrack the generation process and mechanism by linking the eruptive parameters, and predict the behavior and fate by linking the environmental conditions.
3. **Marine particle monitoring**. Marine particles are a ubiquitous and important component in the marine environment, and comprise bio-derived (e.g., phytoplankton) and nonbio-derived (e.g., minerals) particles. Current researches adopted various optical sensors to study the spatial and temporal variability of concentration and size distribution of suspended marine particles^33,34^. However, classification and characterization of specific marine particle assemblages (e.g., marine snow^35^) is always a challenge. The dynamic image analysis and particle morphomics described here may have the potential ability to discriminate different marine particle assemblages based on their distinctive morphological nature. Particle morphomics can also benefit the study on the ultimate supply, transport, and fate of marine particles.
4. **Activated sludge characterization**. The activated sludge is the critical component in a biological process of wastewater treatment plant, and its morphology is correlated with its physical characteristics (e.g., settling velocity^36^), biological characteristics (e.g., microbial community^37^), pollutant removal efficiency^38^, and dewaterability^39^. The previous studies focused more on sludge size impact and ignored the shape influence. The particle morphomics based on image analysis can greatly improve sludge morphological characterization by combining size and shape descriptors and better explain the underlying mechanism of the morphology-dependent sludge properties. Moreover, the discrimination of different particle assemblages (e.g., flocs and filaments^40^) by morphomics analysis can be conducive to evaluation of sludge performance.

## Conclusions

This paper has proposed the concept of particle morphomics, and demonstrated its potential in exploring the morphological diversity and the intrinsic relations with morphological descriptors, evaluating the sample similarity and clustering the samples based on their morphological characteristics. Like other omics, multivariate statistical analyses such as PCoA, RDA and clustering analysis are the useful tools for morphome data mining. Moreover, the particle morphomics may play a greater role in biogeochemical and environmental studies when combing with external factors. Considering the wide use of dynamic image analysis (SI Sheet 2), particle morphomics has wide prospects, and can expand and deepen the current researches related to particle morphology.

## Methods

### Particle sampling and pretreatment

The particle sampling took place in March-May, 2017. Samples 1–7 are sediments, of which 1–5 were collected along the Yangtze River in Shanghai City, and 6 and 7 were collected in a small river in Xinghua City. Samples 8 and 9 are agricultural soils and were collected in the edge of the Yangtze River; samples 10 and 11 are urban soils and were collected at a construction site. Each sediment/soil sample of approximately 1 kg was collected by either a stainless steel grab or an Ekman dredge. Samples 12–14 are dusts, of which 12 was collected with a brush at the windowsill of a student dormitory at Fudan University, and 13 and 14 were collected at the ground near a building materials factory. The detailed sampling information and characteristics of particle samples are described in SI Table S1. The collected samples were air-dried at room temperature, and the biological materials and rocks were removed. After passing through a 5 mesh (5 mm) sieve, particle samples were kept in polypropylene containers at ambient temperature before analysis.

### Sieving for particle size distribution

For traditional sieving, an air-dried sample of approximately 500 g were successively passed through a series of sieves, i.e., 10 mesh (2 mm), 18 mesh (1 mm), 35 mesh (0.5 mm), 65 mesh (0.25 mm), 150 mesh (0.1 mm), and 300 mesh (0.05 mm). The volume of each sieve fraction was measured and used for determination of particle size distribution.

### QICPIC analysis

QICPIC analysis was conducted at a wet dispersing systems with deionized (DI) water as the carrier liquid. Approximately 0.1–1 g of a particle sample was dispersed in 500 mL DI water to make the injecting liquor, which passed through a flow cuvette (10 mm × 30 mm) with a rate of 3 mL/s during the measurement. A high-speed camera with the detection range of 11–22528 μm was employed to obtained the dynamic images of particles with a frame rate of 40 Hz in 90–120 s. For achieving high-resolution digital images of individual particles, the optical concentration of particle flow was kept within 0.5–1% during each measurement.

### Data processing

The morphological data were processed by the WINDOX 5 software. Each particle image was stored, and four morphological descriptors, i.e., equivalent diameter, sphericity, aspect ratio and convexity, were calculated. For fiber-like particles (aspect ratio ≤0.25, sphericity ≤0.25 and convexity ≤0.9), the fiber length, diameter, and elongation were also determined. The detailed definitions of these morphological descriptors are described in SI Text S3. Furtherly, the volume distribution of each morphological descriptor was constructed by using 33 size classes (6.21–79525.52 μm) or 100 shape factor classes (0–1). A series of morphology distribution parameters were calculated including expectation (mean value), SD (standard deviation), kurtosis and skewness, and the calculated equations are provided in SI Text S4.

### Morphomics analysis

The morphological diversity was estimated by Simpson and Shannon indices, which were calculated by the following equations D = 1 − ∑p*p and H = −∑p*Ln(p), respectively. p (%) is the relative volume abundance of each morphological descriptor class. The bivariate correlations among morphology distribution parameters and diversity indices were estimated by Pearson correlation analysis using SPSS 13.0 for Windows (IBM, Armonk, New York, USA). The two-tailed test was used to compute the statistical significance of the correlation coefficient. The *p* value of < 0.01 indicated a significant correlation. Before Pearson correlation analysis, the data normality was checked by Kolmogorov-Smirnov test. Network analysis was carried out using the software Cytoscape (http://www.cytoscape.org) with input Pearson correlation coefficient to explore the associations between morphology distribution parameters and diversity indices. Redundancy analysis (RDA) was executed using Canoco 4.5 for Windows^41^ to investigate effects of morphology distribution parameters on the diversity indices. Forward selection based on the Monte Carlo permutation test (n = 499) was conducted to rank the most important distribution parameters that explained the particle morphological diversity. The heatmap constructed by R software (www.R-project.org) with the pheatmap package was used to illustrate the particle morphology distribution in each sample. Clustering of samples was conducted by the PRIMER 5.0 software (Primer-E Ltd., Plymouth, UK) using the mode of group average based on Bray-Curtis similarity. PCoA analysis based on the particle morphology distribution was performed to compare the differences of samples using the software PRIMER 5.0. Discrete analysis was conducted to reveal the dispersion of particle morphology at each morphological class among the measured samples with OriginPro 8 software (OriginLab Corporation, Northampton, MA, USA).

## Supplementary information

Supporting Information

## Data Availability

The data of this study are available on request from the corresponding author (J.F.).
