# Supplementary material for: Particle morphomics by high-throughput dynamic image analysis
Source: Sci Rep. 2019 Jul 3;9:9591. doi: 10.1038/s41598-019-46062-6 (PMC6610128; doi:10.1038/s41598-019-46062-6)
Supplement: Supplementary file 1 — Supporting Information [file 41598_2019_46062_MOESM1_ESM.pdf]

Supporting Information for

Particle morphomics by high-throughput dynamic image analysis

Youmin Sun, Zhengqing Cai, Jie Fu*

Department of Environmental Science & Engineering, Fudan University, Shanghai 200438, China

* Correspondence and requests for materials should be addressed to J.F. (email: jiefu@fudan.edu.cn)

Index for Supporting Information

Supporting Texts:

Text S1. Measurement principle of QICPIC system …………………………………. 3

Text S2. Workflow of extracting data by WINDOX 5 …………………………….. 4-6

Text S3. Definitions of morphological descriptors …………………………………... 7

Text S4. Calculations of morphology distribution parameters ……………………….. 8

Supporting Tables:

Table S1. Sampling information for particle samples ………………………………... 9

Supporting Figures:

Fig. S1. Comparison of dynamic image analysis and traditional sieving …………… 10

Fig. S2. Average distribution curves of particle size and shape factor ……………… 11

Fig. S3. Discrete analysis of particle morphology distribution ……………………... 12

Fig. S4. Typical fiber-like particle images in samples 1, 8 and 12 ………………….. 13

Supporting Excels:

Sheet 1. Pearson correlation coefficient matrix of particle morphology distribution parameters and diversity indices …………………………………………………….. 14

Sheet 2. Application of dynamic image analysis on particle size and shape measurement ……………………………………………………………………….... 15

Text S1. Measurement principle of QICPIC system

The QICPIC particle size and shape analyzer is an instrument, which can directly measure and analyze the particle size and shape of a large number of fast moving particles. Its measurement is based on the principle of optical imaging, and the following figure present the diagram of particle image measurement. When measuring, the pulsed light emitted from the high-frequency pulsed light source passes through the beam expander to obtain parallel pulsed light, which irradiates on the dispersed single particle in the test area; through a special optical imaging system, the clear image of each particle at the orthogonal orientation of the projection direction is obtained. After a large number of image data are processed by computer, the single particle close-up image, or the morphological information and distribution of all the statistical particles in a sample can be obtained.

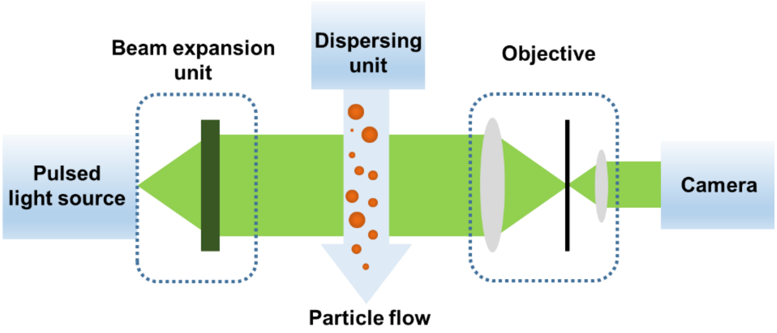

Fig. Diagram of QICPIC measurement system.

The particle size range of QICPIC measurement is from 1 to 20000 μm. The stroboscopic technology uses pulsating light source. The time of each pulse is less than 1×10^{-9} s, and the stroboscopic rate of the lens is about 109 times/s. The high-speed camera system has a resolution of 1024×1024 pixels and a gray scale of 256. Even particles with a moving speed of up to 100 m/s can be clearly imaged. The QICPIC measurement employs the high-speed camera system CMOS, which is capable of shooting 500 images per second, a single-winding double-stranded cable with 1.25 GByte/s of transmission rate, a specially designed PCI interface, and a workstation with dual-core processing technology. Therefore, it can realize the real-time transmission of a large number of test data and images, and obtain tens of thousands to millions of particle images in a very short time, which ensures the density and total amount of test particles, and achieves the well representative results.

Text S2. Workflow of extracting data by WINDOX 5

WINDOX 5 the special software for QICPIC measurement and analysis, which comprises of multi-purpose program groups (packages) for sensor control, real-time storage and processing of high-throughput images, database management, and data output. During the QICPIC measurement, the high-throughput images will be stored real-timely in standard Database as an FDB file. The image data of every single particle including X-coordination, Y-coordination and number of set (black) pixels are encoded as 16 bit values in hexadecimal coding, as shown in the example below.

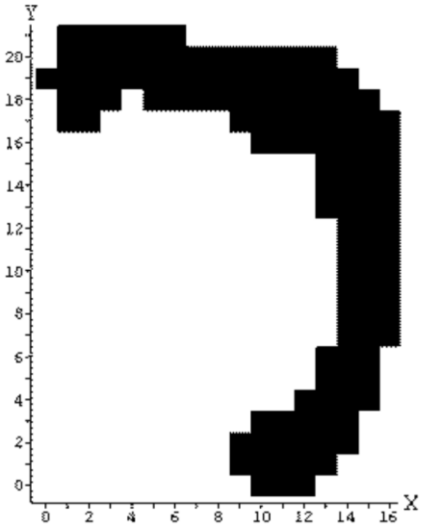

Data	X	Y	Pixel number
0A00 0000 0300	10	0	3
0900 0100 0500	9	1	5
0900 0200 0600	9	2	6
0A00 0300 0500	10	3	5
0C00 0400 0400	12	4	4
0D00 0500 0300	13	5	3
0D00 0600 0300	13	6	3
0E00 0700 0300	14	7	3
0E00 0800 0300	14	8	3
0E00 0900 0300	14	9	3
0E00 0A00 0300	14	10	3
0E00 0B00 0300	14	11	3
0E00 0C00 0300	14	12	3
0D00 0D00 0400	13	13	4
0D00 0E00 0400	13	14	4
0D00 0F00 0400	13	15	4
0A00 1000 0700	10	16	7
0900 1100 0800	9	17	8
0500 1200 0B00	5	18	11
0100 1100 0200	1	17	2
0100 1200 0300	1	18	3
0000 1300 0F00	0	19	15
0100 1400 0D00	1	20	13
0100 1500 0600	1	21	6

Fig. Example of particle image and encoded data.

After the QICPIC measurement, we could choose the calculation properties such as equivalent diameter, sphericity, aspect ratio and convexity (Text S3) in the Work Page, and perform the calculation. The calculation results including morphological descriptors and morphology distribution parameters (Text S4) are integrated in the FDB file. The Application Program provides a programming language, whose grammar is similar to BASIC language, to retrieve and export the morphological data. In Application Program, we can retrieve the morphological data from FDB file by using the statement "JOURNAL" with the function of returning a report of the current measurement data as a string of characters, and a series of Template Commands (see table below). The statement "DATAEXPORT" is used to export the image analysis data into the CSV file.

Table. Template Commands list used in the present study.

Command	Description	Unit
@DDEV	Name of dispersing system	
@MRANGE	Measuring range	
@MDATE	Date of measurement	
@MTIME	Time of measurement	
@MCOMMENT	Complete measurement comment	
@VERMEAS	Version number of WINDOX measuring software	
@PNAME	Product name	
@CPNUM	Number of particles included in the current evaluation	
@C.OPT	Optical concentration	%
@xi.(p,n)	Linear interpolated particle size, with Qn=p%<br>p: percentage, p in [0,100]; n: quantity measure	µm
@VMD.(n)	Volume mean diameter; n: quantity measure	µm
@M.SV	Volume specific surface area	m^2/cm^3
@dmx.(n)	Particle size at the maximum of the linear distribution density; n: quantity measure	µm
@dmxg.(n)	Particle size at the maximum of the logarithmic distribution density; n: quantity measure	µm
@xstdd.(n)	Standard deviation of the particle size distribution<br>n: quantity measure	µm
@SKEW.(n)	Skewness of a size distribution; n: quantity measure	µm
@RRSB.D(n)	RRSB Fineness parameter d’; n: quantity measure	
@RRSB.N(n)	RRSB Fineness parameter n; n: quantity measure	
@RRSB.R2(n)	Regression quality of an RRSB fit<br>n: quantity measure	
@RRSB.S(n)	Standard deviation of the RRSB fit<br>n: quantity measure	
@xexp.(n)	Statistical expectation of the particle size distribution<br>n: quantity measure	µm
@NX	Number of classes of a size distribution	
@x.(k)	Upper limit of particle size class k<br>k: particle size class, k out of {1,...,@NX}	µm
@Q.(k,n)	Cumulative distribution value at the upper particle size limit of class k<br>k: particle size class, k out of {1,...,@NX}<br>n: quantity measure	%
@R.(k,n)	Residue distribution (1-Qn)<br>k: particle size class, k out of {1,...,@NX}<br>n: quantity measure	%
@dQ.(k,n)	Fraction (percentage in size class)<br>k: particle size class, k out of {1,...,@NX}<br>n: quantity measure	%
@q.(k,n)	Linear distribution density	1/mm

	k: particle size class, k out of {1,...,@NX}<br>n: quantity measure	
@xgm.(k)	Geometric mean value of the particle size class k<br>k: particle size class , k out of {1,...,@NX}	µm
@qx.(k,n)	Logarithmic distribution density<br>k: particle size class, k out of {1,...,@NX}<br>n: quantity measure	
@SD.NS	Number of classes of the shape factor distribution	
@SC.S(k,s)	Mean shape factor of the particles in size class k<br>k: ($1 \leq k \leq 1000$); s: calculation mode	
@SD.dmS(s,n)	Shape factor at the maximum of the shape factor distribution density<br>s: calculation mode; n: quantity measure	
@SD.EXC(s,n)	Kurtosis Excess of a size distribution<br>s: calculation mode; n: quantity measure	
@SD.Q(k,s,n)	Cumulative distribution value for shape factor class k<br>k: ($1 \leq k \leq 1000$); s: calculation mode<br>n: quantity measure	%
@SD.q(k,s,n)	Linear distribution density value for shape factor class k ($1 \leq k \leq 1000$)<br>s: calculation mode; n: quantity measure	
@SD.R(k,s,n)	Residue distribution value for shape factor class k<br>k: ($1 \leq k \leq 1000$); s: calculation mode<br>n: quantity measure	%
@SD.S(k,s)	Upper limit of shape factor class k used for the calculation of the distribution ($1 \leq k \leq 1000$)<br>s : calculation mode	
@SD.Sexp(s,n)	Statistically expected value of the shape factor distribution<br>s: calculation mode; n: quantity measure	
@SD.SKEW(s,n)	Skewness of a size distribution<br>s: calculation mode; n: quantity measure	
@SD.Sm(k,s)	Mean value of the shape factor for class k, ($1 \leq k \leq 1000$); s: calculation mode	
@SD.Sstdd(s,n)	Standard deviation of the shape factor distribution<br>s: calculation mode; n: quantity measure	

Wherever in this list, the parameter “n” is mentioned, and describes the quantity measure (0=number, 1=length, 2=area, 3=volume) of the distribution; the parameter “s” is calculation mode for the shape factor (1=sphericity, 2=aspect ratio, 3=convexity).

Text S3. Definitions of morphological descriptors

The equivalent diameter is defined as the diameter of a circle with the same area as the projection area of the particle. The sphericity is the ratio of the perimeter of the equivalent circle to the real perimeter of the particle. The aspect ratio is the ratio of minimal to the maximal Feret diameter of the particle. The convexity is defined as the ratio of the projection area and the area of the convex hull of the particle. The length of a fiber is defined as the longest direct path from one end to another within the particle contour without loops or deviations. The diameter of a fiber is calculated by dividing the projection area by the sum of all lengths of the branches of the fiber. The elongation is the ratio of diameter and length of a fiber.

Text S4. Calculations of morphology distribution parameters

Expectation:

$$E(x) = \int x f(x) dx \qquad \text{(S1)}$$

where x is the morphological descriptor value (i.e., equivalent dimeter, sphericity, aspect ratio or convexity)

Standard deviation:

$$\sigma = \sqrt{\frac{1}{n}\sum_{i=1}^{n}(x_i - \bar{x})^2} \qquad \text{(S2)}$$

where n is the number of size or shape factor class, and $\bar{x}$ is the mean value of a specific descriptor.

Kurtosis:

$$\gamma 2 = \frac{m_4}{m_2^2} - 3 = \frac{\frac{1}{n}\sum_{i=1}^{n}(x_i - \bar{x})^4}{\left(\frac{1}{n}\sum_{i=1}^{n}(x_i - \bar{x})^2\right)^2} - 3 \qquad \text{(S3)}$$

where m_4 is the fourth central moment, and m_2 is the variance.

Skewness:

$$Skew(x) = \frac{1}{n-1}\sum_{i=1}^{n}\frac{(x_i - \bar{x})^3}{\sigma^3} \qquad \text{(S4)}$$

Table S1. Sampling information for particle samples.

No.	Type	Location	Description
1	Sediment	E121°30'29", N31°23'53"	The sample was collected from a wetland park located downstream of the Yangtze River near the estuary. It was comprised of gravels and a small amount of fine particles with a lighter color. No plants grew on it.
2	Sediment	E121°30'30", N31°23'50"	Sampling location was near that of No. 1 sample. The sample was comprised of fine particles with a darker color, and contained numeral fine plant roots.
3	Sediment	E121°18'48", N31°31'0"	The sample was collected at the embankment of Yangtze River near the Baosteel Reservoir. It was comprised of sand and silt particles with a darker color. No plants grew on it.
4	Sediment	E121°18'48", N31°31'0"	The sample was collected from the reeds near the sampling location of No. 3 sample, and had a similar character with No. 3 sample.
5	Sediment	E121°46'5", N31°13'12"	The sample was collected from a small harbor in the estuary of Yangtze River. It was comprised of spongy fine particles with a lighter color. Some reeds were grown on it.
6	Sediment	E119°58'59", N32°53'6"	The sample was collected at the bank of a small river with a gentle flow in Xinghua, Jiangsu. The sample was comprised of particles of various sizes with a black color. Some weeds were grown on it.
7	Sediment	E119°58'58", N32°53'6"	The sample was collected at the central riverway of the same river of collecting No. 6 sample. The sample had a similar character with No. 6 sample but without plants growing on it.
8	Soil	E121°20'44", N31°29'11"	The sample was collected at a paddy field in the suburban area of Baoshan Dist., Shanghai. The field has been reclaimed for a long time with a high fertility. It was about to spring plant when sampling.
9	Soil	E121°45'47", N31°12'54"	The sample was collected from a rape field at Sanjia Port in Pudong Dist. of Shanghai, and comprised of silt and clay particles with a darker color.
10	Soil	E121°29'50", N31°20'25"	The sample was an urban soil and collected at a construction site to be developed in Yangpu Dist. of Shanghai. The sample was withdrawn from top layers, and comprised of varying sizes of particles with a moderate color. The sample was potential backfill.
11	Soil	E121°29'50", N31°20'25"	The sample was the subsoil of No. 10 sample with a higher density and a light yellow color. The sample was an old and compacted urban soil.
12	Dust	E121°29'29", N31°18'7"	The sample was collected at the windowsill of a student dormitory in the Handan Campus of Fudan University with a light grey color.
13	Dust	E121°17'10", N31°8'1"	The sample was collected at the ground close to the residential area. A dust point source was a nearby building materials factory.
14	Dust	E121°17'10", N31°8'1"	The sample was collected at a nearby site close to the sampling location of No. 13 sample but more close to the building materials factory. The sample has a similar character with No. 13 sample.

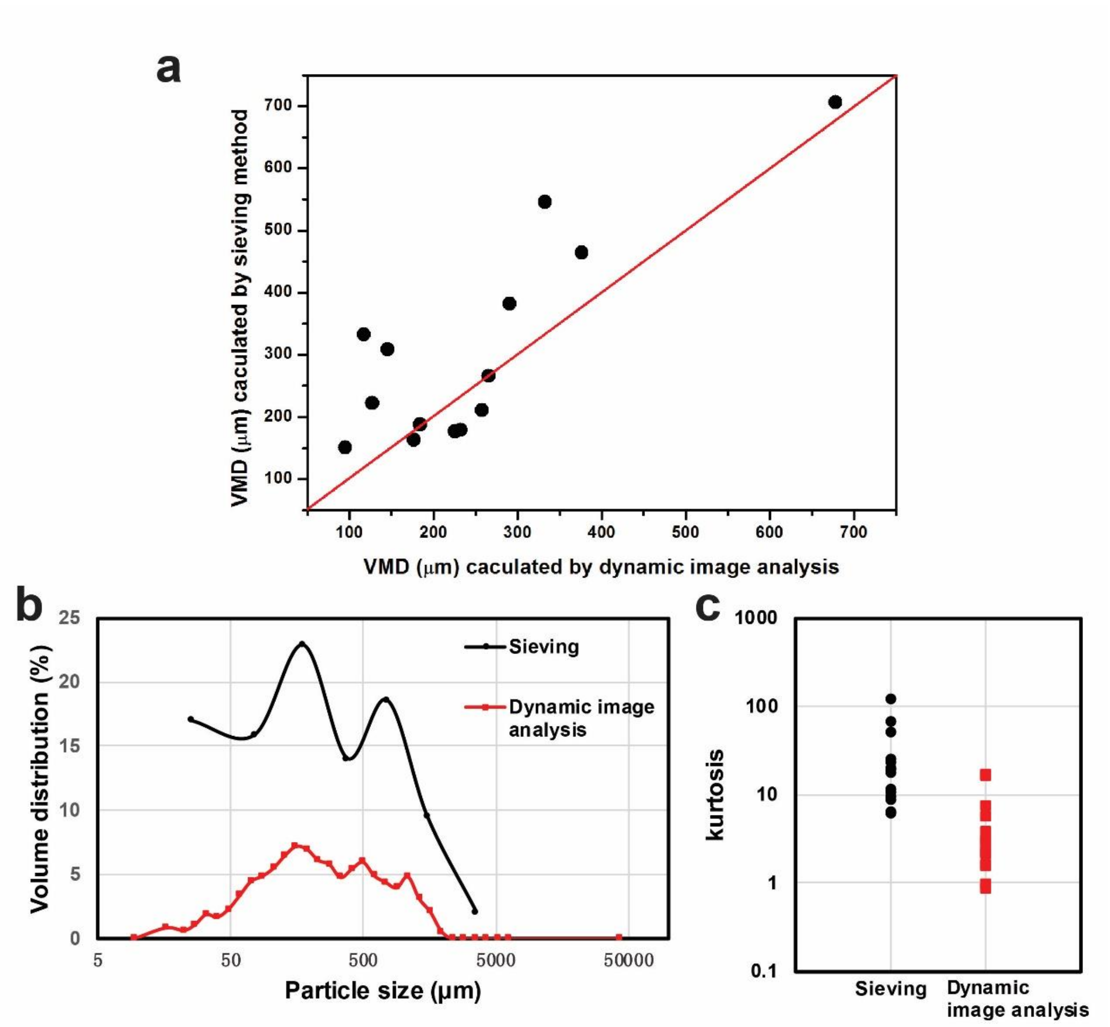

Fig. S1. Comparison of dynamic image analysis and traditional sieving. (a) Plot of VMD calculated by dynamic image analysis versus the value calculated by sieving method. **(b)** Particle size distribution curves of No. 6 sample obtained by dynamic image analysis and sieving method. **(c)** Statistic of kurtosis of particle size distribution obtained by dynamic image analysis and sieving method.

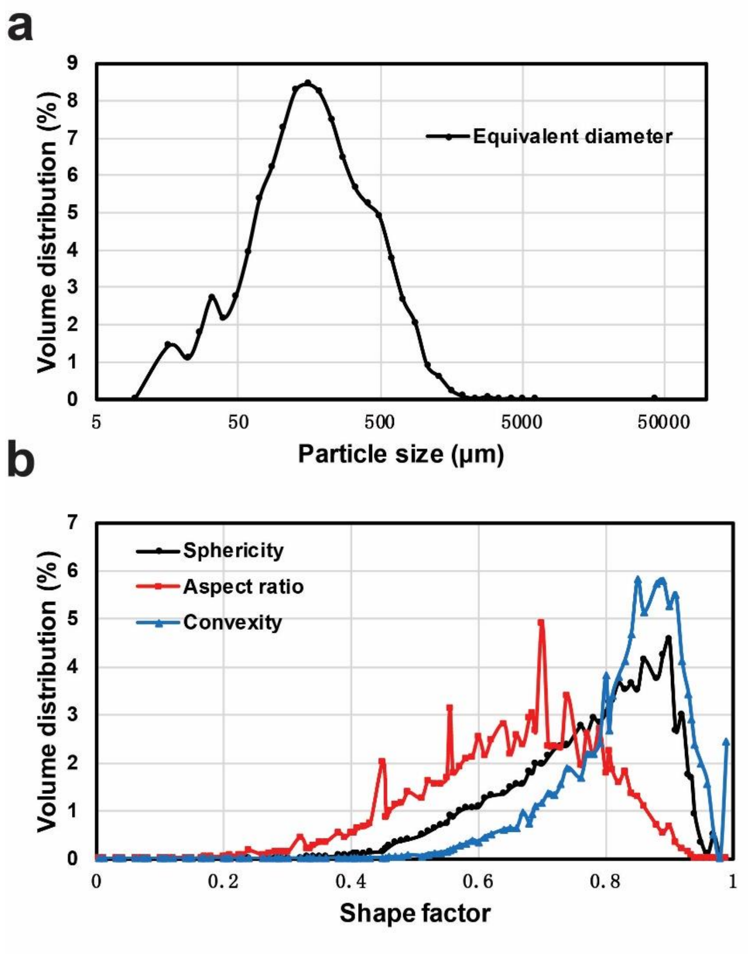

Fig. S2. Average distribution curves of particle size (a) and shape factor (b) of the measured 14 samples.

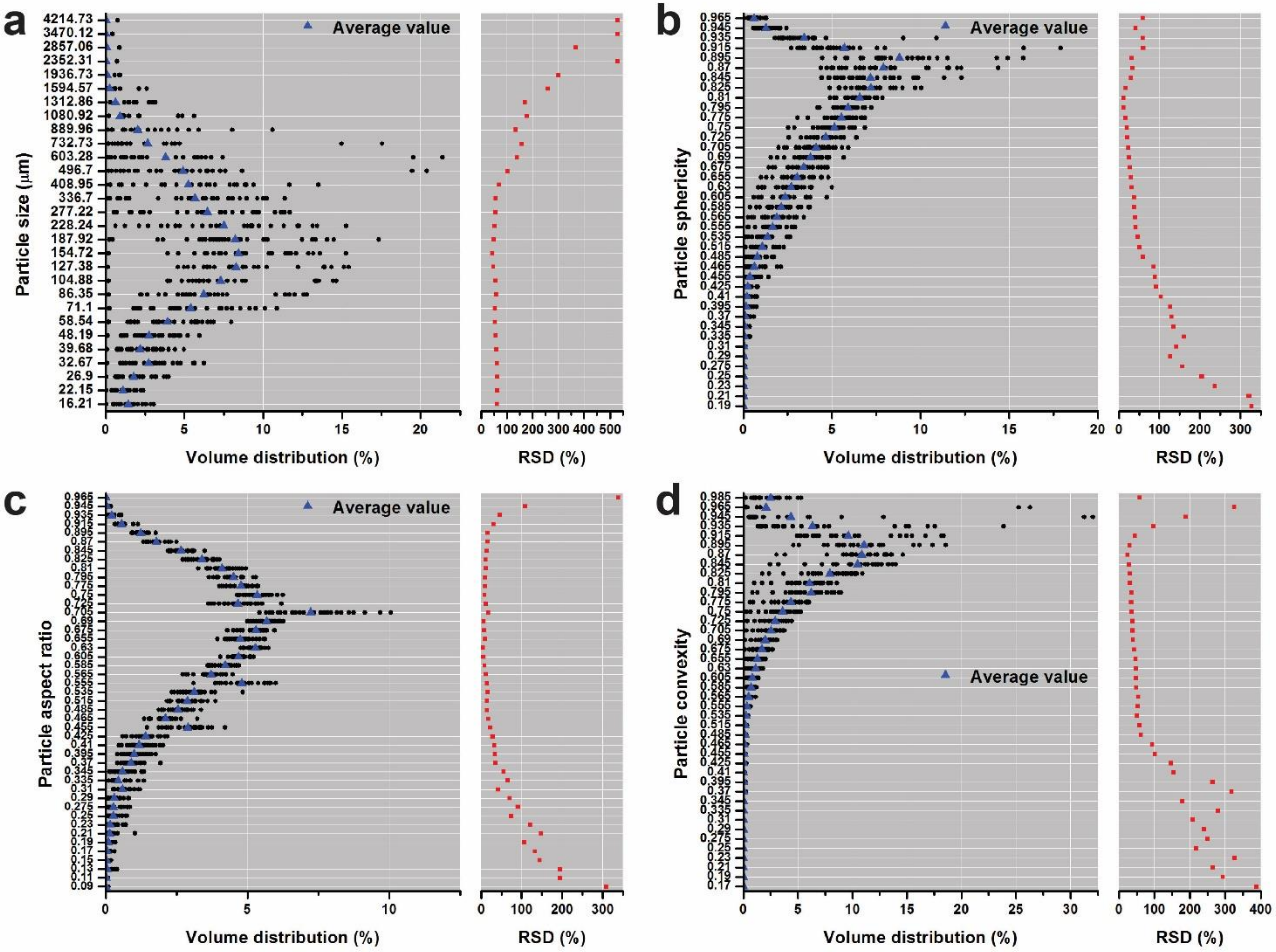

Fig. S3. Discrete analysis of particle morphology distribution at each size or shape class for the measured samples: (a) size, (b) sphericity, (c) aspect ratio, and (d) convexity.

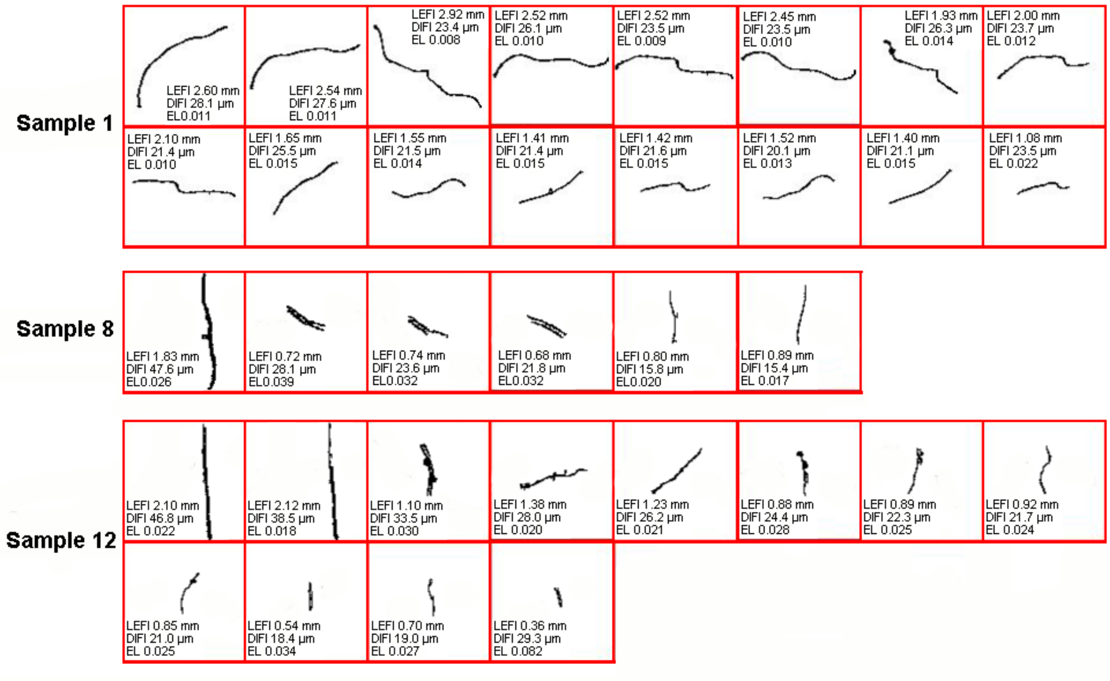

Fig. S4. Typical fiber-like particle images in samples 1 (sediment), 8 (soil) and 12 (dust).

Pearson correlation coefficient matrix of particle morphology distribution parameters and diversity indices

		Size						Sphericity						Aspect ratio						Convexity					
		**VMD**	**SD**	**Kurtosis**	**Skewness**	**Simpson**	**Shannon**	**VMS**	**SD**	**Kurtosis**	**Skewness**	**Simpson**	**Shannon**	**VMA**	**SD**	**Kurtosis**	**Skewness**	**Simpson**	**Shannon**	**VMC**	**SD**	**Kurtosis**	**Skewness**	**Simpson**	**Shannon**
**Size**	**VMD**	1																							
	**SD**	0.912(**)	1																						
	**Kurtosis**	0.358	0.227	1																					
	**Skewness**	0.488(**)	0.399(*)	0.964(**)	1																				
	**Simpson**	-0.363	-0.027	-0.649(**)	-0.596(**)	1																			
	**Shannon**	-0.192	0.156	-0.581(**)	-0.489(**)	0.979(**)	1																		
**Sphericity**	**VMS**	0.353	0.062	0.446(*)	0.386(*)	-0.690(**)	-0.689(**)	1																	
	**SD**	-0.387(*)	-0.118	-0.442(*)	-0.396(*)	0.615(**)	0.590(**)	-0.920(**)	1																
	**Kurtosis**	0.698(**)	0.466(*)	0.371	0.416(*)	-0.549(**)	-0.467(*)	0.841(**)	-0.856(**)	1															
	**Skewness**	-0.609(**)	-0.37	-0.375(*)	-0.405(*)	0.596(**)	0.533(**)	-0.907(**)	0.825(**)	-0.935(**)	1														
	**Simpson**	-0.660(**)	-0.408(*)	-0.487(**)	-0.498(**)	0.670(**)	0.601(**)	-0.916(**)	0.900(**)	-0.957(**)	0.950(**)	1													
	**Shannon**	-0.569(**)	-0.293	-0.483(**)	-0.472(*)	0.695(**)	0.645(**)	-0.951(**)	0.942(**)	-0.934(**)	0.938(**)	0.990(**)	1												
**Aspect ratio**	**VMA**	-0.223	-0.470(*)	0.232	0.065	-0.535(**)	-0.658(**)	0.679(**)	-0.582(**)	0.338	-0.418(*)	-0.455(*)	-0.533(**)	1											
	**SD**	0.166	0.448(*)	-0.265	-0.124	0.625(**)	0.732(**)	-0.715(**)	0.629(**)	-.382(*)	0.474(*)	0.513(**)	0.602(**)	-.900(**)	1										
	**Kurtosis**	-0.31	-0.27	-0.307	-0.343	0.233	0.155	-0.192	0.169	-0.113	0.234	0.263	0.267	0.235	0.013	1									
	**Skewness**	0.324	0.187	0.326	0.352	-0.395(*)	-0.343	0.363	-0.357	0.271	-0.376(*)	-0.415(*)	-0.443(*)	0.012	-0.331	-0.842(**)	1								
	**Simpson**	0.589(**)	0.706(**)	0.034	0.217	0.158	0.331	-0.309	0.2	0.07	-0.035	0.011	0.098	-0.747(**)	0.665(**)	-0.342	0.156	1							
	**Shannon**	0.489(**)	0.675(**)	-0.075	0.107	0.332	0.494(**)	-0.510(**)	0.397(*)	-0.099	0.178	0.215	0.314	-0.877(**)	0.850(**)	-0.197	-0.057	0.948(**)	1						
**Convexity**	**VMC**	0.826(**)	0.616(**)	0.396(*)	0.468(*)	-0.557(**)	-0.441(*)	0.775(**)	-0.790(**)	0.938(**)	-0.922(**)	-0.935(**)	-0.902(**)	0.2	-0.277	-0.308	0.428(*)	0.284	0.082	1					
	**SD**	-0.713(**)	-0.493(**)	-0.501(**)	-0.553(**)	0.652(**)	0.545(**)	-0.680(**)	0.796(**)	-0.817(**)	0.756(**)	0.857(**)	0.849(**)	-0.224	0.374(*)	0.363	-0.540(**)	-0.259	-0.038	-0.872(**)	1				
	**Kurtosis**	0.867(**)	0.643(**)	0.494(**)	0.562(**)	-0.685(**)	-0.561(**)	0.637(**)	-0.653(**)	0.858(**)	-0.770(**)	-0.831(**)	-0.781(**)	0.156	-0.189	-0.146	0.242	0.269	0.144	0.889(**)	-0.820(**)	1			
	**Skewness**	-0.868(**)	-0.691(**)	-0.356	-0.465(*)	0.533(**)	0.396(*)	-0.622(**)	0.639(**)	-0.884(**)	0.844(**)	0.829(**)	0.773(**)	-0.056	0.12	0.197	-0.284	-0.425(*)	-0.261	-0.943(**)	0.827(**)	-0.935(**)	1		
	**Simpson**	-0.835(**)	-0.591(**)	-0.530(**)	-0.584(**)	0.723(**)	0.610(**)	-0.739(**)	0.760(**)	-0.911(**)	0.841(**)	0.917(**)	0.880(**)	-0.235	0.308	0.241	-0.372	-0.203	-0.041	-0.933(**)	0.899(**)	-0.973(**)	0.923(**)	1	
	**Shannon**	-0.795(**)	-0.542(**)	-0.495(**)	-0.550(**)	0.718(**)	0.611(**)	-0.759(**)	0.804(**)	-0.917(**)	0.860(**)	0.926(**)	0.902(**)	-0.257	0.362	0.279	-0.441(*)	-0.204	-0.015	-0.946(**)	0.943(**)	-0.942(**)	0.925(**)	0.987(**)	1

* correlation is significant at the 0.05 level (2-tailed); ** correlation is significant at the 0.01 level (2-tailed); particle sample number (including duplicates) = 28.

Abbreviation: VMD, volume mean diameter; VMS, volume mean sphericity; VMA, volume mean aspect ratio; VMC, volume mean convexity; SD, standard deviation.

Application of dynamic image analysis on particle size and shape measurement

Particle type	Analysis instrument	Objective	Reference
Granular solids	QICPIC	Study on particle wettability controlled by particle morphology	Eur J Soil Sci, 2018, 69: 698-709
Blend of lactose and starch	Self-made device	Real-time feedback control of particle size	Int J Pharm, 2018, 547: 360-367
Wood pellet	Camsizer	Wood pellet milling tests	Fuel Proc Technol, 2018, 173: 89-102
Solid inorganic fertilizers	Self-made device	Development of a new dynamic image analysis system	Comput Electron Agr, 2018, 147: 146-157
Grober quarze	Bettersizer S3 Plus	Development of a new particle morphology analysis instrument	Chem Ing Tech, 2018, 90: 419-426
Pharmaceutical powder	SEM and Malvern Mastersizer 3000	Study on effect of fractal particle on dynamic flow of excipients	J Pharm Innov, 2018, 13: 15-26
Talc mineral	Micromeritics	Quality control of the products of talc mineral	Particul Sci Technol, 2018, 36: 332-339
Gas-solid mixture	Self-made device	Presentation of a new measurement technique for gas-solid mixture	J Environ Manage, 2017, 203: 942-949
Wood particles	X-ray CT	Comparative study on 2D image analysis and X-ray CT	Mater Test, 2017, 59: 829-836
Infused particle	QICPIC	Evaluation subvisible particles during drug infusion	Sci Rep, 2017, 7, 9404
Volcanic ash	QICPIC	Characterization of volcanic ash of a recent eruptive event	Measurement, 2017, 104: 336-346
Alumina	Camsizer	Characterization of fused and sintered alumina aggregates	Ceram Int, 2017, 43: 4252-4262
Concrete aggregate fines	X-ray CT	Measurement of particle size distribution and specific surface area	Adv Powder Technol, 2017, 28: 706-720
Concrete aggregate fines	AnaTec, FPA	Comparative study on 2D and 3D shape analysis	Powder Technol, 2017, 309: 110-125
Coal	Self-made device	Investigation of the particle behavior during coal screening	Powder Technol, 2017, 306: 88-95
Microparticles of cardiac extracellular matrixs	QICPIC	Characterization of microparticles	J Mater Sci-Mater M, 2016, 27: 120
Wood plastic composites	QICPIC	Evaluation on particle degradation	Compos Part A-Appl S, 2016, 84: 464-471
Coal	Micromeritics	Modeling on particle size distribution	Fuel Proc Technol, 2016, 143: 100-109
Minitablets	Camsizer XT	Determination of the coating thickness	Int J Pharm, 2018, 495: 347-353
Metal powder	Camsizer XT	Characterization and control of powder properties	JOM, 2015, 67: 549-554
Calcite particles	Micromeritics	Characterization of calcite particles	Int J Miner Process, 2014, 133: 83-90
Concrete aggregates	AnaTec, FPA	Characterization of pore structure, specific surface, and morphology	Cement Concrete Comp, 2014, 54: 2-16
Coal	Micromeritics	Shape analysis of particle	Fuel Proc Technol, 2014, 126: 350-358
Flax fibers	QICPIC	Study on effect of extruder elements on fiber dimensions	J Appl Polym Sci, 2014, 131: 40435
Pharmaceutical granules	FlashSizer3D	Real-time at-line analysis of granules	Eur J Pharm Biopharm, 2012, 82: 429-436
Ceramic hydroxyapatite	FPIA-3000, Malvern	Monitoring of the degradation process	Biotechnol J, 2012, 7: 1288-1296
D-mannitol needle crystals	Micromeritics	Demonstration of particle shape affecting fracture mechanism	Pharm Res, 2012, 29: 2806-2816
Cellobiose octaacetate needle crystals	QICPIC	Characterization of needle crystals	Analyst, 2012, 137: 118-125
Dairy powders	QICPIC	Study on rehydration properties of dairy powders	Int Dairy J, 2011, 21: 462-469
Drug precipitates	XPT®-C particle analyser	Monitoring of drug precipitation	J Pharm Pharmacol, 2011, 63: 333-341
Pharmaceutical granules	XPT®-CV	On-line dynamic image analysis	J Pharm Innov, 2010, 5: 100-108
Pharmaceutical granules	XPT® particle analyser	Monitoring of pharmaceutical dry milling process	Int J Pharm, 2010, 391: 107-114
Organic crystals	Camsizer	Characterization of crystal shape	Part Part Syst Char, 2009, 26: 171-178
D-mannitol crystals	QICPIC	Characterization of crystal shape	Cryst Growth Des, 2009, 9: 4907-4911
Loess	ADIA	Investigation on heterogeneity of Brabantian loess	Quatern Int, 2009, 198: 195-203
Activated sludge floc	CIS-100	Quantification of the activated sludge floc size distribution	Water Sci Technol, 2009, 60: 1857-1867
Pharmaceutical excipient particles	QICPIC	Characterization of pharmaceutical excipient particles	Int J Pharm, 2008, 361: 150-157
Sedimentary grains	ADIA	Size and shape analysis	Part Part Syst Char, 2007, 23: 381-387
Drug pellets	Camsizer	Study on swelling behavior in acid and buffer during drug release	Pharm Dev Technol, 2007, 12, 285-296
Latex and glass beeds	CIS-100	Comparison on different particle analysis techniques	Part Part Syst Char, 2006, 23: 145-153
PVC powders	Self-made device	Development of a new analysis technique	Part Part Syst Char, 2006, 23: 165-169
Drug dosage	Camsizer	Measurement of mean size of multiparticluate sample	Pharm Dev Technol, 2006, 11, 403-408
Glass beads	RapidVUE	Comparison on different particle analysis techniques	Powder Technol, 2003, 132: 145-153
Foams	Self-made device	Modeling flotation performance	Int J Miner Process, 2002, 67: 79-99
